# The plasmacytoid dendritic cell paradox in cancer: impaired type I interferon responses in a nucleic acid–rich tumor microenvironment

**DOI:** 10.3389/fimmu.2026.1847012

**Published:** 2026-07-02

**Authors:** Aliki Vasilakou, Séverine Loizon, Maxime Dubois, Paôline Laurent, Vanja Sisirak, Dorothée Duluc

**Affiliations:** Univ. Bordeaux, CNRS, ImmunoConcEpT, UMR 5164, Bordeaux, France

**Keywords:** cancer, microenvironment, nucleic acid, plasmacytoid dendritic cells, type I IFN

## Abstract

Plasmacytoid dendritic cells (pDCs) are specialized immune cells best known for their ability to produce large amounts of type I interferons (IFN-I) upon nucleic acid sensing through endosomal Toll-like receptors (TLR)-7/8 and 9. In tumors, the accumulation of nucleic acids (NAs) deriving from extensive cancer cell death would be expected to activate pDCs and induce IFN-I–mediated anti-tumor responses. However, tumor-infiltrating pDCs typically exhibit impaired IFN-I production and instead acquire immunosuppressive properties, revealing a paradox between NAs availability and pDC function within the tumor microenvironment (TME). In this review, we examine current knowledge and explore the mechanisms that may constrain IFN-I responses by pDCs, including chronic stimulation, alterations in TLR signaling and limited accessibility of tumor-derived NAs. We discuss key open questions regarding how tumor-associated signals may impair pDC responsiveness and contribute to their dysfunction, the nature and immunostimulatory potential of tumor-derived NAs, and their intracellular delivery. By highlighting these unresolved questions, we propose conceptual frameworks to better understand pDC biology in cancer and identify strategies to restore their IFN-I–mediated antitumor functions.

## 1 Introduction

Dendritic cells (DCs) are central regulators of innate and adaptive immunity, and are broadly classified into conventional DCs (cDCs), including the cDC1 and cDC2 subsets, and plasmacytoid DCs (pDCs) (1). More recently, transitional DCs (tDCs) have been described as a hybrid subset related to pDCs (2, 3). PDCs specialize in producing type I and III interferons (IFN-I and IFN-III) (1, 4–6). These responses are triggered by nucleic acids (NA) sensing through the endosomal Toll-like receptors (TLR)7 and TLR9, and depend on IFN regulatory factor (IRF)-7 activation (7). Initially characterized in viral infection, pDCs rapidly recognize viral NAs and orchestrate antiviral immune responses (8–10), but are also implicated in sterile settings such as autoimmunity and cancer via self-NA recognition (11).

Tumors are sites of cellular stress, genomic instability, and extensive cell death occurring either spontaneously or following cytotoxic therapies, leading to the release of damage-associated molecular patterns (DAMPs), a process termed immunogenic cell death (ICD) (12). Anti-tumor immunity is initiated and amplified by these DAMPs, particularly NAs, which engage immune sensors to trigger IFN-I pathways critical for tumor control (13–15). IFN-I, including IFN-α and IFN-β, are crucial for anti-tumor immunity [reviewed in (16)], as defective IFN-I signaling accelerates tumor progression, whereas its induction enhances responses to therapy (16–19). Although multiple cell types, including cDCs and cancer cells, contribute to IFN-I production, they do so through distinct NA sensing pathways. cDC1 detects tumor-derived DNA through cytosolic cyclic GMP-AMP synthase (cGAS) – stimulator of interferon genes (STING) signaling, leading to IFN-β production and T cell cross-priming, while pDCs recognize NA by endosomal TLRs (TLR7/8 for RNA and TLR9 for DNA recognition), and produce substantial amounts of IFN-α (7, 20).

Given their tumor infiltration, high IFN-I production capacity, and NA abundance in tumors, pDCs would be expected to play a key role in anti-tumor immunity (1, 21–23). Paradoxically, tumor-infiltrating pDCs often fail to mount robust IFN-I responses, instead adopting an immunosuppressive phenotype (21, 24–26). This suggests that tumors actively restrain pDC IFN-I responses. Understanding these mechanisms is therefore critical to define the role of pDCs in cancer and harness their therapeutic potential.

In this review, we examine how the tumor microenvironment (TME) constrains pDC-mediated IFN-I responses, focusing on tumor-mediated drivers of pDC exhaustion, tumor-derived NA properties, and their sensing and uptake by pDCs.

## 2 Functional reprogramming of pDCs in cancer

Given this discrepancy between their intrinsic IFN-I–producing capacity and their dysfunctional state in tumors, a key question is how the TME reprograms pDCs. PDCs are frequently associated with poor prognosis across multiple cancer types, including melanoma, lung, breast, ovarian and gastric cancers (21, 24–29). These studies are supported by *in vivo* evidence showing that antibody-mediated pDC depletion delays tumor growth in murine models (30–33). However, this association is not universal, as beneficial associations with tumor-infiltrating lymphocytes and improved survival have also been reported in follicular lymphoma and colorectal carcinoma (34–36), and in some models, pDC depletion instead promotes tumor growth (36). Together, these observations indicate that pDC functions are TME-dependent.

We hypothesize that pDC prognostic impact is largely determined by their functional state, particularly their IFN-producing capacity. Consistent with this idea, IFN-α production by pDCs correlates with favorable outcomes in several cancers. In melanoma, although pDC function was generally impaired, IFN-α-producing capacity of pDCs upon ex vivo TLR stimulation correlates with good prognosis (28). Similarly, in colorectal cancer, pDCs expressing nuclear IRF-7 localize near granzyme B^+^ CD8 T cells and associate with better prognosis, suggesting a role in anti-tumor immunity through IRF7-mediated IFN-I production (35). Conversely, impaired IFN-I production by tumor-infiltrating pDCs is observed in tumor types where pDCs are associated with poor prognosis (26, 37–41).

These findings support a model in which pDC functional status, rather than mere presence, determines their role in tumor progression. The TME may drive this reprogramming through CXCL12-mediated sequestration, and the release of cytokines such as VEGF, TNF-α, TGF-β and IL-10 that impair pDC maturation and activation (21). Consequently, pDCs promote Th2 polarization and regulatory T cell (Treg) expansion (27, 37, 42, 43). Interestingly, impaired IFN-I production was associated with acquisition of immunosuppressive functions in tumor-infiltrating pDCs (44). Whether specific tumor niches preferentially support immunosuppressive versus IFN-producing pDC states remains unclear.

This raises the central question: which mechanisms within the TME impair IFN-I production in pDCs, despite their intrinsic responsiveness to NA sensing?

## 3 Mechanisms of functional alterations of tumor-infiltrating pDCs

### 3.1 Alterations in the IRF-7 pathway and TLR expression

Several studies have shown that immunosuppressive TME factors, including TNFα and TGFβ, directly impair IFN-I production (38, 45–47). These effects are mediated, at least in part, through downregulation of TLR expression and inhibition of IRF-7 expression or nuclear translocation (45, 48–50). However, whether this reflects a stable defect or a reversible state imposed by the TME remains unclear. To address this, several studies have examined the ex vivo responsiveness of pDCs to TLR7 and TLR9. In multiple tumor types, such as breast cancer, non-small cell lung cancer, or head and neck cancer, reduced responsiveness to TLR stimulation has been reported, indicating persistent impairment (38, 41, 49, 51). However, this dysfunction is not uniform across tumor types. In melanoma, IFN-I production following TLR7 activation is preserved, with only partial impairment observed for TLR9 stimulation (27). Similarly, in ovarian cancer, tumor-associated pDCs retain partial responsiveness to TLR7 ligands (26). These observations suggest that defects in IFN-I production may not solely reflect loss of TLR signaling capacity but also dynamic, TLR-specific regulation by the TME, with TLR7 and TLR9 likely govern by distinct regulatory mechanisms.

### 3.2 Chronic stimulation and progressive pDC exhaustion

Another hypothesis is that pDC dysfunction in cancer progressively arises from chronic TME stimulation. Physiologically, the IFN-I–producing capacity of pDCs requires tight regulation to prevent excessive inflammation (7, 52). In the context of viral infections, sustained stimulation can lead to pDC exhaustion, characterized by reduced IFN-I production and functional impairment (53). For example, persistent lymphocytic choriomeningitis virus (LCMV) infection illustrates how continuous TLR7 stimulation decreases IFN-I production. Moreover, prolonged exposure to IFN-I alters bone marrow dynamics, limiting the generation of functionally competent pDCs (53). These findings indicate that chronic stimulation may reduce pDC numbers and reprogram their functional state (54), including through metabolic alterations (55). Interestingly, in other contexts of chronic inflammation such as obesity, pDCs retain their capacity to produce IFN-I and are drivers of the disease (56, 57), highlighting that chronic exposure does not universally induce exhaustion.

Chronic inflammation is a hallmark of cancer, but whether chronic exposure to tumor-derived NAs drives pDC exhaustion remains an open question. Interestingly, prolonged IFN signaling can promote immunosuppression and resistance to immune checkpoint blockade (58, 59). Moreover, the intensity of TLR stimulation can differentially shape immune responses, as illustrated by studies showing that high doses of TLR9 ligands promote indoleamine 2,3-dioxygenase (IDO) production and Treg activation, whereas lower doses favor Th1 responses. Although these observations were not specifically demonstrated in pDCs, they suggest that sustained or dysregulated IFN-I signaling may contribute to the tolerogenic phenotype of tumor-associated pDCs (60–62). Finally, TLR9 has a reported regulatory role in autoimmune lupus (63); while primarily attributed to B cells (64), whether similar effects occur in pDCs remains unknown, adding complexity to the NA–pDC–IFN-I axis in cancer.

It is therefore reasonable to propose that, at early cancer stages, pDCs contribute to immune surveillance through NA sensing and IFN-I production, whereas sustained exposure to the TME drives progressive functional exhaustion. Some clinical observations support this model. In colorectal cancer, high pDC density correlates with early tumor stages and improved survival, and these cells express nuclear IRF-7 and localize near T lymphocytes. In contrast, advanced stages are associated with reduced pDC infiltration and poorer outcomes (35). These findings suggest that pDC abundance and function evolve during tumor progression. However, mouse studies often report that pDC depletion prior to tumor implantation reduces tumor growth and improves survival, suggesting a pro-tumoral role (30–33). This apparent discrepancy may reflect limitations of transplantation models, which bypass early tumor development. The lack of longitudinal and physiologically relevant models remains a major limitation to define the temporal role of pDCs in cancer. In addition, depletion strategies lack specificity, as models selectively targeting pDCs without affecting related populations such as tDCs have only recently emerged (65). This raises the possibility that some effects attributed to pDCs may involve other DC subsets.

### 3.3 Is pDC functional status acquired in the TME reversible?

Recent findings suggest that, despite chronic stimulation and TME-induced suppression, tumor-associated pDCs retain functional plasticity and that their apparent exhaustion may not be irreversible. For instance, IFN-III can reprogram tumor-associated pDCs from an immunosuppressive toward a more immunostimulatory phenotype, restoring IFN-I production and enhancing their ability to induce effector T cell responses (66). Preclinical studies have also shown that TLR7/8 and TLR9 agonists used as adjuvants of anti-cancer therapies can enhance anti-tumor immune responses and improve outcomes (67). Although TLR9 agonists have shown limited efficacy as monotherapy, their use in combination therapies appears more promising (68, 69). Similarly, the TLR7 agonist imiquimod has demonstrated anti-tumor activity, notably in skin cancers, partly through increased IFN-I production, cytotoxicity and immune cell recruitment (67). However, while some studies suggest that TLR ligands can act through pDC activation (70–73), only a few have demonstrated a causal role using pDC depletion approaches, either genetic or antibody-based (74, 75). Therefore, whether these therapeutic effects critically depend on pDCs remains to be further clarified.

Collectively, these findings indicate that tumor-associated pDCs retain functional responsiveness to TLR7 and TLR9 stimulation. This raises a key mechanistic question: beyond intrinsic signaling defects, could limitations in NA availability, immunostimulatory potential, or intracellular access explain defective pDC activation in situ?

## 4 NAs within the TME and pDC functions

### 4.1 Limiting factors of pDC activation: NAs quantity and immunostimulatory potential?

Tumor cells continuously release DNA and RNA into the TME through apoptosis, necrosis, and micronuclei formation (23). Tumor-derived RNAs exist in multiple forms and can be packaged inside extracellular vesicles (76). Tumor-derived DNA is primarily genomic and mitochondrial and can be found complexed with proteins, within apoptotic bodies and extracellular vesicles/microparticles or as extrachromosomal DNA (23, 77, 78). Despite this abundance, endogenous tumor RNA or DNA rarely triggers IFN-I production by pDCs *in vivo*, highlighting a disconnect between NA release and pDC activation. While TLR9 can sense DNA, most anti-tumor effects have been demonstrated with synthetic TLR9 agonists, such as CpG oligodeoxynucleotides, suggesting that endogenous tumor DNA may not efficiently activate TLR9 in pDCs (79, 80). Conversely, tumor-derived DNA can access the cytosol of cDCs, activating cGAS–STING signaling and leading to IFN-I production and T cell priming (13, 20, 81). However, cGAS depletion does not always limit spontaneous tumor growth, indicating that DNA presence alone may be insufficient for robust immune activation (15, 82). These findings highlight differences in sensing between cell types and suggest that limited NA abundance, accessibility, or immunostimulatory potential may constrain pDC activation in the TME.

Extracellular nucleases could limit the availability and immunostimulatory potential of tumor-derived NAs. DNASE1 and DNASE1L3 are the two main secreted DNASEs digesting extracellular DNA (83). In autoimmunity, DNASE1L3, primarily secreted by DCs, digests complex host DNA released during cell death to prevent aberrant activation of NA sensors (84). In cancer, DNASE1L3 is downregulated in multiple tumor types (85). Intriguingly, its deficiency in colon cancer models correlates with reduced expression of IFN-I–related genes at later stages (86). Moreover, tumor DNA can originate from neutrophil extracellular traps (NETs), which are generally pro-tumoral and favor metastasis (87). NETs activity is limited by DNASE1 and DNASE1L3, thereby limiting tumor growth (88, 89). Collectively, these observations indicate that extracellular nucleases may restrict both the quantity and the immunostimulatory potential of tumor-derived NAs, potentially limiting pDC activation in the TME.

Although spontaneous NA release may be limiting, therapeutic interventions can increase both the abundance and functional potential of extracellular NAs (90). Cell free (cf) DNA levels are elevated in cancer patients and transiently increase following cytotoxic therapies such as chemotherapy (CT) and radiotherapy (RT) (91–93). However, this cfDNA pool derives from both tumor and non-tumor cells, and may not accurately reflect local concentrations within the TME (23). In addition to increasing NA quantity, therapies can alter their immunostimulatory properties. For example, CT can induce the release of CA-rich repeat domains, which strongly engage cGAS on DCs and promote anti-tumor immunity, whereas CA-poor DNA preferentially activates AIM2 and may drive immunosuppressive responses (94). Interestingly, a recent study highlights that RNA properties influences TLR7/8 activation (95). These observations suggest that both the abundance and biochemical characteristics of tumor-derived NAs influence their capacity to activate immune responses, including potential effects on pDCs, although direct evidence remains limited.

Importantly, while cGAS activation in cDC1 is well documented following CT and RT, and can drive IFN-I production and T cell priming (96), it remains unclear whether TLR9 in tumor-infiltrating pDCs is similarly engaged. Some *in vivo* studies indicate that tumor control following cisplatin or doxorubicin therapy is partially dependent on TLR9 signaling (97). However, whether this effect is mediated by pDCs or other TLR9-expressing cell types has not been definitively established. Similarly, TLR7 agonists can enhance anti-tumor immunity in therapeutic settings (67), yet evidence that endogenous tumor RNA engages TLR7 in pDCs *in vivo* remains limited.

These findings raise an important question: why can cDC1 respond to tumor-derived DNA via cGAS–STING, while pDCs, which rely on endosomal TLR7/9, appear less responsive? Differences in phagocytic capacity, intracellular routing, and NA accessibility between these DC subsets likely contribute. cDC1 efficiently transfer phagosomal DNA to the cytosol for cGAS sensing (81), whereas pDCs are less phagocytic and may rely on additional cofactors or specific delivery mechanisms to access endosomal TLRs. Altogether, limitations in NA quantity, immunostimulatory potential, or intracellular delivery may restrict pDC activation, even when extracellular NAs are abundant.

### 4.2 Could NA internalization be the limiting step? Lessons from autoimmunity

The ligand access to TLR7/9 is tightly restricted as these receptors are confined to endosomal compartments to prevent inappropriate recognition of self-NAs (98, 99). Spatial restriction is critical for immune tolerance, as forced membrane TLR9 expression leads to the recognition of naked host DNA, which is poorly immunostimulatory unless efficiently delivered to endosomes, highlighting the importance of NA transport mechanisms (100). In contrast to cDC1, pDCs appear less efficient to phagocyte dead cells and NA uptake, potentially increasing their reliance on extracellular cofactors to mediate endosomal delivery. Autoimmune diseases provide key insights into how this barrier can be overcome. The positive charges of cationic cofactors including LL-37, CXCL4, CXCL10, CXCL12, and IL-26, enable NA protection, and along with NA-binding proteins such as HMGB1, promote NA sensing through better uptake and endosomal delivery (101–116). Thus, they convert otherwise inert self-NAs into potent immunostimulatory ligands.

In cancer, however, whether similar delivery systems occur remains unclear. Tumor-derived NAs may be abundant, particularly following ICD (117), and DNA-binding cofactors are present in tumors [e.g., released by dying cells, neutrophils, platelets, or cancer cells (118–120)]; however, it remains poorly defined whether these cofactors effectively facilitate NA access to pDC endosomes. For example, although LL-37 was shown to enhance CpG-mediated anti-tumor responses (121), whether this effect is mediated by pDCs remains unresolved. In addition, HMGB1 in cancer displays context-dependent functions as it can enhance TLR9 signaling through RAGE-mediated trafficking (113–115), but has also been reported to inhibit pDC activation or promote tolerogenic phenotypes in certain tumor settings (122, 123). Additional regulatory layers may further restrict NA sensing in tumors. For instance, HMGB1-mediated DNA internalization can be inhibited through its interaction with TIM-3, an inhibitory receptor frequently upregulated on tumor-infiltrating DCs, suggesting a potential mechanism by which tumors limit TLR-driven activation (124, 125). Moreover, while CXCL4 can bind self-DNA and activate pDCs in autoimmune contexts, in colorectal cancer, tumor CXCL4 expression increases following 5-Fluorouracil CT, which inhibits T cell responses and promotes tumor growth (126). Similarly, IL-26- and NET-associated DNA, while capable of promoting NA sensing in inflammatory settings, have been linked to pro-tumoral inflammation and immunosuppression in cancer, highlighting their context-dependent effects (118). Beyond their role in facilitating uptake, NA-binding cofactors may also influence the stability and extracellular lifespan of nucleic acid complexes. Although largely unexplored in the context of pDC dysfunction, the highly proteolytic TME may further limit the availability and immunostimulatory potential of NA-cofactor complexes (127). Several cofactors implicated in extracellular NA sensing, including LL-37, IL-26, and HMGB1, bind NAs and facilitate their delivery to endosomal TLRs. Beyond promoting uptake, these interactions may also protect extracellular NAs from nuclease-mediated degradation. Indeed, IL-26-DNA complexes and LL-37-associated DNA exhibit enhanced resistance to DNASE digestion, thereby prolonging the availability of immunostimulatory ligands and promoting pDC activation (110, 128–130). Consequently, proteolytic degradation or remodeling of NA-binding cofactors within the TME could destabilize these complexes, expose previously protected NAs to extracellular nucleases, and reduce the persistence of immunostimulatory ligands available for pDC sensing. Supporting this concept, citrullination of LL-37 by peptidyl arginine deiminases (PADs) destabilizes its interaction with NET-associated DNA, thereby limiting pDC activation (129, 131). However, the proteolytic processing of HMGB1 by neutrophil elastase (NE) enhanced its DNA binding affinity, which could instead increase the endocytosis of NAs and therefore DNA sensing by pDCs (132). Although direct evidence in tumors is lacking, the combined activity of proteases and nucleases may critically influence the stability and immunostimulatory capacity of extracellular NA complexes.

Interestingly, in viral infections, pDCs efficiently sense infected cells through the transfer of NA-containing material, bypassing the need for free NA diffusion (133). Whether similar cell-cell transfer mechanisms operate within the TME remains speculative. Altogether, insufficient or dysregulated NA internalization, rather than NA availability alone, may limit pDC activation in tumors. This may explain why synthetic TLR agonists efficiently activate pDCs, whereas evidence for activation by endogenous tumor-derived NAs remains limited in the TME. Importantly, most mechanistic insight into NA delivery derives from autoimmunity or other DC subsets, and direct evidence in tumor-infiltrating pDCs remains limited.

## 5 Conclusion

**Figure 1:**
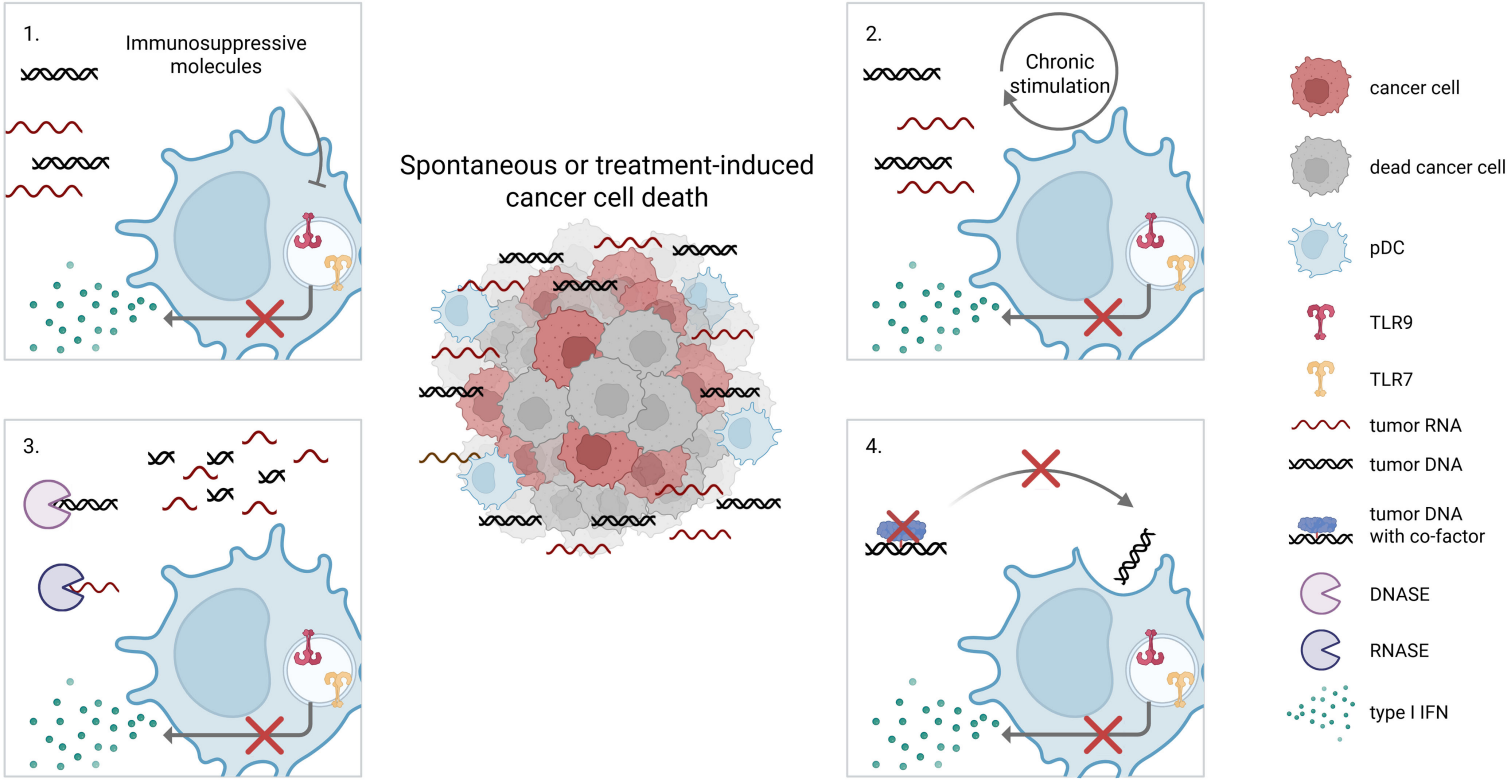
Why are type I IFN responses by pDCs impaired? We hypothesize several non-mutually exclusive mechanisms that could explain why pDCs have impaired IFN-I-production in the tumor microenvironment: 1. Immunosuppressive TME: The TME may inhibit pDC responsiveness to TLR7/9 activation. 2. Chronic activation: Persistent NA sensing within the TME may drive pDC exhaustion and reduce responsiveness to TLR7/9. 3. Extracellular nucleases: Secreted nucleases may degrade tumor-derived NAs, diminishing their immunostimulatory capacity. 4. Lack of DNA-binding cofactors: Absence of essential DNA-binding cofactors may impair tumor DNA internalization, contributing to defective TLR7/9 activation. Created in BioRender. https://BioRender.com/vbiqsb9.

PDCs play a central role in immune responses due to their unique capacity to sense NAs and secrete IFN-I. While NAs from dying tumor cells are expected to activate pDCs and promote antitumor immunity, tumor-infiltrating pDCs often fail to mount robust responses and instead acquire a tolerogenic state. This paradox suggests that tumors restrain pDC IFN-I responses through multiple, non-mutually exclusive mechanisms (**Figure 1**).

First, both defective and chronic IFN-I signaling may promote tumor progression, highlighting the need for tightly regulated pDC activation. Defining when and how pDCs transition toward tolerogenic states remains critical, particularly using genetic tumor models capturing early and late stages of progression, and new pDC-deficient mouse models may help address this gap. Second, pDC TLR functionality may be partially preserved, as tumor-associated pDCs respond to synthetic TLR7/9 agonists, suggesting that limitations extend beyond signaling to NA availability, immunostimulatory potential, and intracellular delivery. Extracellular nucleases may further restrict NA sensing, although their role in tumors remains unclear. Third, autoimmunity studies show that cofactors bind NAs, protect them, and promote endosomal TLR activation; whether similar mechanisms occur in tumors is unclear, raising the possibility that insufficient cofactor-mediated NA delivery contributes to impaired pDC activation.

Collectively, these findings support a model in which impaired or actively restricted NA internalization, rather than defective TLR signaling alone, constitutes a major limitation for pDC activation in cancer—one that synthetic TLR ligands can bypass. Elucidating how tumor-derived NAs are processed, protected, and trafficked *in vivo* will be essential to resolve this paradox and may provide strategies to restore pDC-mediated antitumor immunity.

## References

[1] Fitzgerald-Bocarsly P Dai J Singh S . Plasmacytoid dendritic cells and type I IFN: 50 years of convergent history. Cytokine Growth Factor Rev. (2008) 19:3–19. doi: 10.1016/j.cytogfr.2007.10.006 18248767 PMC2277216

[2] Idoyaga J Ni H Maqueda‐Alfaro RA . Bridging pDCs and cDCs: The identity of transitional dendritic cells. Immunol Rev. (2025) 336:e70070. doi: 10.1111/imr.70070 41275417 PMC12640671

[3] Sulczewski FB Maqueda-Alfaro RA Alcántara-Hernández M Perez OA Saravanan S Yun TJ . Transitional dendritic cells are distinct from conventional DC2 precursors and mediate proinflammatory antiviral responses. Nat Immunol. (2023) 24:1265–80. doi: 10.1038/s41590-023-01545-7 37414907 PMC10683792

[4] Sato A Ohtsuki M Hata M Kobayashi E Murakami T . Antitumor activity of IFN-λ in murine tumor models. J Immunol. (2006) 176:7686–94. doi: 10.4049/jimmunol.176.12.7686 16751416

[5] Tang B Liu Z Xiong H Zhang J Dai J . IFN-λ: Unleashing its potential in disease therapies from acute inflammation regulation to cancer immunotherapy. Immunology. (2025) 176:197–214. doi: 10.1111/imm.13954 40421666

[6] Yin Z Dai J Deng J Sheikh F Natalia M Shih T . Type III IFNs are produced by and stimulate human plasmacytoid dendritic cells. J Immunol. (2012) 189:2735–45. doi: 10.4049/jimmunol.1102038 22891284 PMC3579503

[7] Reizis B Bunin A Ghosh HS Lewis KL Sisirak V . Plasmacytoid dendritic cells: Recent progress and open questions. Annu Rev Immunol. (2011) 29:163–83. doi: 10.1146/annurev-immunol-031210-101345 21219184 PMC4160806

[8] Ngo C Garrec C Tomasello E Dalod M . The role of plasmacytoid dendritic cells (pDCs) in immunity during viral infections and beyond. Cell Mol Immunol. (2024) 21:1008–35. doi: 10.1038/s41423-024-01167-5 38777879 PMC11364676

[9] Dupuis S Jouanguy E Al-Hajjar S Fieschi C Al-Mohsen IZ Al-Jumaah S . Impaired response to interferon-alpha/beta and lethal viral disease in human STAT1 deficiency. Nat Genet. (2003) 33:388–91. doi: 10.1038/ng1097 12590259

[10] Duncan CJA Randall RE Hambleton S . Genetic lesions of type I interferon signalling in human antiviral immunity. Trends Genet. (2021) 37:46–58. doi: 10.1016/j.tig.2020.08.017 32977999 PMC7508017

[11] Lande R Gilliet M . Plasmacytoid dendritic cells: Key players in the initiation and regulation of immune responses. Ann N Y Acad Sci. (2010) 1183:89–103. doi: 10.1111/j.1749-6632.2009.05152.x 20146710

[12] Tesniere A Apetoh L Ghiringhelli F Joza N Panaretakis T Kepp O . Immunogenic cancer cell death: A key-lock paradigm. Curr Opin Immunol. (2008) 20:504–11. doi: 10.1016/j.coi.2008.05.007 18573340

[13] Woo SR Fuertes MB Corrales L Spranger S Furdyna MJ Leung MYK . STING-dependent cytosolic DNA sensing mediates innate immune recognition of immunogenic tumors. Immunity. (2014) 41:830–42. doi: 10.1016/j.immuni.2014.10.017 25517615 PMC4384884

[14] Sistigu A Yamazaki T Vacchelli E Chaba K Enot DP Adam J . Cancer cell–autonomous contribution of type I interferon signaling to the efficacy of chemotherapy. Nat Med. (2014) 20:1301–9. doi: 10.1038/nm.3708 25344738

[15] Deng L Liang H Xu M Yang X Burnette B Arina A . STING-dependent cytosolic DNA sensing promotes radiation-induced type I interferon-dependent antitumor immunity in immunogenic tumors. Immunity. (2014) 41:843–52. doi: 10.1016/j.immuni.2014.10.019 25517616 PMC5155593

[16] Holicek P Guilbaud E Klapp V Truxova I Spisek R Galluzzi L . Type I interferon and cancer. Immunol Rev. (2024) 321:115–27. doi: 10.1111/imr.13272 37667466

[17] Schreiber RD Old LJ Smyth MJ . Cancer immunoediting: Integrating immunity’s roles in cancer suppression and promotion. Science. (2011) 331:1565–70. doi: 10.1126/science.1203486 21436444

[18] Vesely MD Kershaw MH Schreiber RD Smyth MJ . Natural innate and adaptive immunity to cancer. Annu Rev Immunol. (2011) 29:235–71. doi: 10.1146/annurev-immunol-031210-101324 21219185

[19] Razaghi A Durand-Dubief M Brusselaers N Björnstedt M . Combining PD-1/PD-L1 blockade with type I interferon in cancer therapy. Front Immunol. (2023) 14:1249330. doi: 10.3389/fimmu.2023.1249330 37691915 PMC10484344

[20] Ng KW Marshall EA Bell JC Lam WL . cGAS–STING and cancer: Dichotomous roles in tumor immunity and development. Trends Immunol. (2018) 39:44–54. doi: 10.1016/j.it.2017.07.013 28830732

[21] Zhou B Lawrence T Liang Y . The role of plasmacytoid dendritic cells in cancers. Front Immunol. (2021) 12:749190. doi: 10.3389/fimmu.2021.749190 34737750 PMC8560733

[22] Kawai T Akira S . The role of pattern-recognition receptors in innate immunity: Update on Toll-like receptors. Nat Immunol. (2010) 11:373–84. doi: 10.1038/ni.1863 20404851

[23] Stejskal P Goodarzi H Srovnal J Hajdúch M van ’t Veer LJ Magbanua MJM . Circulating tumor nucleic acids: Biology, release mechanisms, and clinical relevance. Mol Cancer. (2023) 22:15. doi: 10.1186/s12943-022-01710-w 36681803 PMC9862574

[24] Jensen TO Schmidt H Møller HJ Donskov F Høyer M Sjoegren P . Intratumoral neutrophils and plasmacytoid dendritic cells indicate poor prognosis and are associated with pSTAT3 expression in AJCC stage I/II melanoma. Cancer. (2012) 118:2476–85. doi: 10.1002/cncr.26511 21953023

[25] Treilleux I Blay JY Bendriss-Vermare N Ray-Coquard I Bachelot T Guastalla JP . Dendritic cell infiltration and prognosis of early stage breast cancer. Clin Cancer Res. (2004) 10:7466–74. doi: 10.1158/1078-0432.CCR-04-0684 15569976

[26] Labidi-Galy SI Sisirak V Meeus P Gobert M Treilleux I Bajard A . Quantitative and functional alterations of plasmacytoid dendritic cells contribute to immune tolerance in ovarian cancer. Cancer Res. (2011) 71:5423–34. doi: 10.1158/0008-5472.CAN-11-0367 21697280

[27] Aspord C Leccia MT Charles J Plumas J . Plasmacytoid dendritic cells support melanoma progression by promoting Th2 and regulatory immunity through OX40L and ICOSL. Cancer Immunol Res. (2013) 1:402–15. doi: 10.1158/2326-6066.CIR-13-0114-T 24778133

[28] Sosa Cuevas E Bendriss-Vermare N Mouret S De Fraipont F Charles J Valladeau-Guilemond J . Diversification of circulating and tumor-infiltrating plasmacytoid DCs towards the P3 (CD80+ PDL1-)-pDC subset negatively correlated with clinical outcomes in melanoma patients. Clin Trans Immunol. (2022) 11:e1382. doi: 10.1002/cti2.1382 35517992 PMC9063720

[29] Labidi-Galy SI Treilleux I Goddard-Leon S Combes JD Blay JY Ray-Coquard I . Plasmacytoid dendritic cells infiltrating ovarian cancer are associated with poor prognosis. Oncoimmunology. (2012) 1:380–2. doi: 10.4161/onci.18801 22737622 PMC3382863

[30] Sorrentino R Morello S Luciano A Crother TR Maiolino P Bonavita E . Plasmacytoid dendritic cells alter the antitumor activity of CpG-oligodeoxynucleotides in a mouse model of lung carcinoma. J Immunol. (2010) 185:4641–50. doi: 10.4049/jimmunol.1000881 20855872

[31] Le Mercier I Poujol D Sanlaville A Sisirak V Gobert M Durand I . Tumor promotion by intratumoral plasmacytoid dendritic cells is reversed by TLR7 ligand treatment. Cancer Res. (2013) 73:4629–40. doi: 10.1158/0008-5472.CAN-12-3058 23722543

[32] Yang LL Mao L Wu H Chen L Deng WW Xiao Y . pDC depletion induced by CD317 blockade drives the antitumor immune response in head and neck squamous cell carcinoma. Oral Oncol. (2019) 96:131–9. doi: 10.1016/j.oraloncology.2019.07.019 31422204

[33] Sawant A Hensel JA Chanda D Harris BA Siegal GP Maheshwari A . Depletion of plasmacytoid dendritic cells inhibits tumor growth and prevents bone metastasis of breast cancer cells. J Immunol. (2012) 189:4258–65. doi: 10.4049/jimmunol.1101855 23018462 PMC3531993

[34] Butsch R Lukas waelti S Schaerer S Braun J Korol D Probst-hensch N . Intratumoral plasmacytoid dendritic cells associate with increased survival in patients with follicular lymphoma. Leukemia Lymphoma. (2011) 52:1230–8. doi: 10.3109/10428194.2011.569619 21599580

[35] Kießler M Plesca I Sommer U Wehner R Wilczkowski F Müller L . Tumor-infiltrating plasmacytoid dendritic cells are associated with survival in human colon cancer. J Immunother Cancer. (2021) 9:e001813. doi: 10.1136/jitc-2020-001813 33762320 PMC7993360

[36] Poropatich K Dominguez D Chan WC Andrade J Zha Y Wray B . OX40^+^ plasmacytoid dendritic cells in the tumor microenvironment promote antitumor immunity. J Clin Invest. (2020) 130:3528–42. doi: 10.1172/JCI131992. PMC7324178 32182225

[37] Faget J Bendriss-Vermare N Gobert M Durand I Olive D Biota C . ICOS-ligand expression on plasmacytoid dendritic cells supports breast cancer progression by promoting the accumulation of immunosuppressive CD4+ T cells. Cancer Res. (2012) 72:6130–41. doi: 10.1158/0008-5472.CAN-12-2409 23026134

[38] Sisirak V Faget J Gobert M Goutagny N Vey N Treilleux I . Impaired IFN-α production by plasmacytoid dendritic cells favors regulatory T-cell expansion that may contribute to breast cancer progression. Cancer Res. (2012) 72:5188–97. doi: 10.1158/0008-5472.CAN-11-3468 22836755

[39] Sorrentino R Terlizzi M Crescenzo VGD Popolo A Pecoraro M Perillo G . Human lung cancer–derived immunosuppressive plasmacytoid dendritic cells release IL-1α in an AIM2 inflammasome-dependent manner. Am J Pathol. (2015) 185:3115–24. doi: 10.1016/j.ajpath.2015.07.009 26506473

[40] Shi W Li X Porter JL Ostrodi DH Yang B Li J . Level of plasmacytoid dendritic cells is increased in non-small cell lung carcinoma. Tumor Biol. (2014) 35:2247–52. doi: 10.1007/s13277-013-1297-7 24136746

[41] Perrot I Blanchard D Freymond N Isaac S Guibert B Pachéco Y . Dendritic cells infiltrating human non-small cell lung cancer are blocked at immature stage. J Immunol. (2007) 178:2763–9. doi: 10.4049/jimmunol.178.5.2763 17312119

[42] Ito T Yang M Wang YH Lande R Gregorio J Perng OA . Plasmacytoid dendritic cells prime IL-10–producing T regulatory cells by inducible costimulator ligand. J Exp Med. (2007) 204:105–15. doi: 10.1084/jem.20061660 17200410 PMC2118437

[43] Pedroza-Gonzalez A Zhou G Vargas-Mendez E Boor PP Mancham S Verhoef C . Tumor-infiltrating plasmacytoid dendritic cells promote immunosuppression by Tr1 cells in human liver tumors. Oncoimmunology. (2015) 4:e1008355. doi: 10.1080/2162402X.2015.1008355 26155417 PMC4485712

[44] Sisirak V Faget J Vey N Blay JY Ménétrier-Caux C Caux C . Plasmacytoid dendritic cells deficient in IFNα production promote the amplification of FOXP3+ regulatory T cells and are associated with poor prognosis in breast cancer patients. Oncoimmunology. (2013) 2:e22338. doi: 10.4161/onci.22338 23482834 PMC3583914

[45] Sisirak V Vey N Goutagny N Renaudineau S Malfroy M Thys S . Breast cancer-derived transforming growth factor-β and tumor necrosis factor-α compromise interferon-α production by tumor-associated plasmacytoid dendritic cells. Int J Cancer. (2013) 133:771–8. doi: 10.1002/ijc.28072 23389942

[46] Demoulin S Herfs M Delvenne P Hubert P . Tumor microenvironment converts plasmacytoid dendritic cells into immunosuppressive/tolerogenic cells: Insight into the molecular mechanisms. J Leukoc Biol. (2013) 93:343–52. doi: 10.1189/jlb.0812397 23136258

[47] Bekeredjian-Ding I Schäfer M Hartmann E Pries R Parcina M Schneider P . Tumour-derived prostaglandin E and transforming growth factor-beta synergize to inhibit plasmacytoid dendritic cell-derived interferon-alpha. Immunology. (2009) 128:439–50. doi: 10.1111/j.1365-2567.2009.03134.x 20067543 PMC2770691

[48] Yang L Li S Chen L Zhang Y . Emerging roles of plasmacytoid dendritic cell crosstalk in tumor immunity. Cancer Biol Med. (2023) 20(10):728–47. doi: 10.20892/j.issn.2095-3941.2023.0241 37817484 PMC10618948

[49] Hartmann E Wollenberg B Rothenfusser S Wagner M Wellisch D Mack B . Identification and functional analysis of tumor-infiltrating plasmacytoid dendritic cells in head and neck cancer. Cancer Res. (2003) 63:6478–87 14559840

[50] Han N Zhang Z Jv H Hu J Ruan M Zhang C . Culture supernatants of oral cancer cells induce impaired IFN-α production of pDCs partly through the down-regulation of TLR-9 expression. Arch Oral Biol. (2018) 93:141–8. doi: 10.1016/j.archoralbio.2018.06.006 29913322

[51] Hack K Reilly L Proby C Fleming C Leigh I Foerster J . Wnt5a inhibits the CpG oligodeoxynucleotide-triggered activation of human plasmacytoid dendritic cells. Clin Exp Dermatol. (2012) 37:557–61. doi: 10.1111/j.1365-2230.2012.04362.x 22607321

[52] Snell LM McGaha TL Brooks DG . Type I interferon in chronic virus infection and cancer. Trends Immunol. (2017) 38:542–57. doi: 10.1016/j.it.2017.05.005 28579323 PMC8059441

[53] Macal M Jo Y Dallari S Chang AY Dai J Swaminathan S . Self-renewal and Toll-like receptor signaling sustain exhausted plasmacytoid dendritic cells during chronic viral infection. Immunity. (2018) 48:730–744.e5. doi: 10.1016/j.immuni.2018.03.020 29669251 PMC5937984

[54] Greene TT Jo Y Zuniga EI . Infection and cancer suppress pDC derived IFN-I. Curr Opin Immunol. (2020) 66:114–22. doi: 10.1016/j.coi.2020.08.001 32947131 PMC8526282

[55] Greene TT Jo Y Chiale C Macal M Fang Z Khatri FS . Metabolic deficiencies underlie reduced plasmacytoid dendritic cell IFN-I production following viral infection. Nat Commun. (2025) 16:1460. doi: 10.1038/s41467-025-56603-5 39920132 PMC11805920

[56] Hannibal TD Schmidt-Christensen A Nilsson J Fransén-Pettersson N Hansen L Holmberg D . Deficiency in plasmacytoid dendritic cells and type I interferon signalling prevents diet-induced obesity and insulin resistance in mice. Diabetologia. (2017) 60:2033–41. doi: 10.1007/s00125-017-4341-0 28660492 PMC6448810

[57] Li C Wang G Sivasami P Ramirez RN Zhang Y Benoist C . Interferon-α-producing plasmacytoid dendritic cells drive the loss of adipose tissue regulatory T cells during obesity. Cell Metab. (2021) 33:1610–1623.e5. doi: 10.1016/j.cmet.2021.06.007 34256015 PMC8350961

[58] Benci JL Xu B Qiu Y Wu TJ Dada H Victor CTS . Tumor interferon signaling regulates a multigenic resistance program to immune checkpoint blockade. Cell. (2016) 167:1540–1554.e12. doi: 10.1016/j.cell.2016.11.022 27912061 PMC5385895

[59] Qiu J Xu B Ye D Ren D Wang S Benci JL . Cancer cells resistant to immune checkpoint blockade acquire interferon-associated epigenetic memory to sustain T cell dysfunction. Nat Cancer. (2023) 4:43–61. doi: 10.1038/s43018-022-00490-y 36646856

[60] Mellor AL Baban B Chandler PR Manlapat A Kahler DJ Munn DH . Cutting edge: CpG oligonucleotides induce splenic CD19+ dendritic cells to acquire potent indoleamine 2,3-dioxygenase-dependent T cell regulatory functions via IFN type 1 signaling1. J Immunol. (2005) 175:5601–5. doi: 10.4049/jimmunol.175.9.5601 16237046

[61] Wingender G Garbi N Schumak B Jüngerkes F Endl E von Bubnoff D . Systemic application of CpG‐rich DNA suppresses adaptive T cell immunity via induction of IDO. Eur J Immunol. (2006) 36:12–20. doi: 10.1002/eji.200535602 16323249

[62] Lemos H Huang L McGaha TL Mellor AL . Cytosolic DNA sensing via the stimulator of interferon genes (STING) adaptor: The yin and yang of immune responses to DNA. Eur J Immunol. (2014) 44:2847–53. doi: 10.1002/eji.201344407 25143264 PMC4197080

[63] Leibler C John S Elsner RA Thomas KB Smita S Joachim S . Genetic dissection of TLR9 reveals complex regulatory and cryptic proinflammatory roles in mouse lupus. Nat Immunol. (2022) 23:1457–69. doi: 10.1038/s41590-022-01310-2 36151396 PMC9561083

[64] Leibler C Thomas KB Josensi C Levack RC Smita S John S . Divergent TIR signaling domains in TLR7 and TLR9 control opposing effects on systemic autoimmunity. J Clin Invest. (2025) 135(21):e189566. doi: 10.1172/JCI189566 40794441 PMC12578402

[65] Valente M Collinet N Vu Manh TP Popoff D Rahmani K Naciri K . Novel mouse models based on intersectional genetics to identify and characterize plasmacytoid dendritic cells. Nat Immunol. (2023) 24:714–28. doi: 10.1038/s41590-023-01454-9 36928414 PMC10063451

[66] Sakref C Saby A Rodriguez C Ardin M Moudombi L Doffin AC . Type III interferon primes pDCs for TLR7 activation and antagonizes immune suppression mediated by TGF-β and PGE2. Nat Commun. (2025) 16:3045. doi: 10.1038/s41467-025-58220-8 40155377 PMC11953300

[67] Rolfo C Giovannetti E Martinez P McCue S Naing A . Applications and clinical trial landscape using Toll-like receptor agonists to reduce the toll of cancer. NPJ Precis Onc. (2023) 7:26. doi: 10.1038/s41698-023-00364-1 36890302 PMC9995514

[68] Dongye Z Li J Wu Y . Toll-like receptor 9 agonists and combination therapies: Strategies to modulate the tumour immune microenvironment for systemic anti-tumour immunity. Br J Cancer. (2022) 127:1584–94. doi: 10.1038/s41416-022-01876-6 35902641 PMC9333350

[69] Su C Kent CL Pierpoint M Floyd W Luo L Williams NT . Enhancing radiotherapy response via intratumoral injection of a TLR9 agonist in autochthonous murine sarcomas. JCI Insight. (2024) 9(14):e178767. doi: 10.1172/jci.insight.178767 39133651 PMC11383182

[70] Aspord C Tramcourt L Leloup C Molens JP Leccia MT Charles J . Imiquimod inhibits melanoma development by promoting pDC cytotoxic functions and impeding tumor vascularization. J Invest Dermatol. (2014) 134:2551–61. doi: 10.1038/jid.2014.194 24751730

[71] Inglefield JR Dumitru CD Alkan SS Gibson SJ Lipson KE Tomai MA . TLR7 agonist 852A inhibition of tumor cell proliferation is dependent on plasmacytoid dendritic cells and type I IFN. J Interferon Cytokine Res. (2008) 28:253–63. doi: 10.1089/jir.2007.0097 18439103

[72] Molenkamp BG van Leeuwen PAM Meijer S Sluijter BJR Wijnands PGJTB Baars A . Intradermal CpG-B activates both plasmacytoid and myeloid dendritic cells in the sentinel lymph node of melanoma patients. Clin Cancer Res. (2007) 13:2961–9. doi: 10.1158/1078-0432.CCR-07-0050 17504997

[73] Sanlorenzo M Novoszel P Vujic I Gastaldi T Hammer M Fari O . Systemic IFN-I combined with topical TLR7/8 agonists promotes distant tumor suppression by c-Jun-dependent IL-12 expression in dendritic cells. Nat Cancer. (2025) 6:175–93. doi: 10.1038/s43018-024-00889-9 39849091 PMC11779648

[74] Drobits B Holcmann M Amberg N Swiecki M Grundtner R Hammer M . Imiquimod clears tumors in mice independent of adaptive immunity by converting pDCs into tumor-killing effector cells. J Clin Invest. (2012) 122:575–85. doi: 10.1172/JCI61034 22251703 PMC3266798

[75] Nierkens S den Brok MH Garcia Z Togher S Wagenaars J Wassink M . Immune adjuvant efficacy of CpG oligonucleotide in cancer treatment is founded specifically upon TLR9 function in plasmacytoid dendritic cells. Cancer Res. (2011) 71:6428–37. doi: 10.1158/0008-5472.CAN-11-2154 21788345 PMC3653311

[76] Valadi H Ekström K Bossios A Sjöstrand M Lee JJ Lötvall JO . Exosome-mediated transfer of mRNAs and microRNAs is a novel mechanism of genetic exchange between cells. Nat Cell Biol. (2007) 9:654–9. doi: 10.1038/ncb1596 17486113

[77] Chan LK Shan J Rodriguez-Fos E Healy ME Leary P Parrotta R . Extrachromosomal circular DNA promotes inflammation and hepatocellular carcinoma development. Sci Adv. (2025) 11:eadw0272. doi: 10.1126/sciadv.adw0272 41105787 PMC12533639

[78] Wang Y Wang M Djekidel MN Chen H Liu D Alt FW . eccDNAs are apoptotic products with high innate immunostimulatory activity. Nature. (2021) 599:308–14. doi: 10.1038/s41586-021-04009-w 34671165 PMC9295135

[79] Miller CL Sagiv-Barfi I Neuhöfer P Czerwinski DK Bertozzi CR Cochran JR . Targeted TLR9 agonist elicits effective antitumor immunity against spontaneously arising breast tumors. J Immunol. (2023) 211:295–305. doi: 10.4049/jimmunol.2200950 37256255 PMC10315437

[80] Conrad C Meller S Gilliet M . Plasmacytoid dendritic cells in the skin: To sense or not to sense nucleic acids. Semin Immunol. (2009) 21:101–9. doi: 10.1016/j.smim.2009.01.004 19250840

[81] Xu MM Pu Y Han D Shi Y Cao X Liang H . Dendritic cells but not macrophages sense tumor mitochondrial DNA for cross-priming through signal regulatory protein α signaling. Immunity. (2017) 47:363–373.e5. doi: 10.1016/j.immuni.2017.07.016 28801234 PMC5564225

[82] Wang H Hu S Chen X Shi H Chen C Sun L . cGAS is essential for the antitumor effect of immune checkpoint blockade. Proc Natl Acad Sci. (2017) 114:1637–42. doi: 10.1073/pnas.1621363114 28137885 PMC5320994

[83] Santa P Garreau A Serpas L Ferriere A Blanco P Soni C . The role of nucleases and nucleic acid editing enzymes in the regulation of self-nucleic acid sensing. Front Immunol. (2021) 12:629922. doi: 10.3389/fimmu.2021.629922 33717156 PMC7952454

[84] Sisirak V Sally B D’Agati V Martinez-Ortiz W Özçakar ZB David J . Digestion of chromatin in apoptotic cell microparticles prevents autoimmunity. Cell. (2016) 166:88–101. doi: 10.1016/j.cell.2016.05.034 27293190 PMC5030815

[85] Deng Z Xiao M Du D Luo N Liu D Liu T . DNASE1L3 as a prognostic biomarker associated with immune cell infiltration in cancer. Onco Targets Ther. (2021) 14:2003–17. doi: 10.2147/OTT.S294332 33776450 PMC7987320

[86] Li W Nakano H Fan W Li Y Sil P Nakano K . DNASE1L3 enhances antitumor immunity and suppresses tumor progression in colon cancer. JCI Insight. (2023) 8(17):e168161. doi: 10.1172/jci.insight.168161 37581941 PMC10544201

[87] Brambilla M Zanichelli A Cancila V Colombo MP Chiodoni C Sangaletti S . Neutrophil extracellular traps in cancer: Immune modulation, therapy resistance, and the dilemma of targeting. Cell Death Dis. (2025) 16:882. doi: 10.1038/s41419-025-08218-3 41360771 PMC12686526

[88] Park J Wysocki RW Amoozgar Z Maiorino L Fein MR Jorns J . Cancer cells induce metastasis-supporting neutrophil extracellular DNA traps. Sci Transl Med. (2016) 8:361ra138. doi: 10.1126/scitranslmed.aag1711 27798263 PMC5550900

[89] Chen D Jin Z Chu H Wu Y Bian Y Yuan T . DNASE1L3-expressing dendritic cells promote CD8+ T cell function and anti-PD-(L)1 therapy efficacy by degrading neutrophil extracellular traps. Cancer Cell. (2025) 43:1758–1775.e8. doi: 10.1016/j.ccell.2025.07.014 40816293

[90] Zitvogel L Apetoh L Ghiringhelli F Kroemer G . Immunological aspects of cancer chemotherapy. Nat Rev Immunol. (2008) 8:59–73. doi: 10.1038/nri2216 18097448

[91] Cheng C Omura-Minamisawa M Kang Y Hara T Koike I Inoue T . Quantification of circulating cell-free DNA in the plasma of cancer patients during radiation therapy. Cancer Sci. (2009) 100:303–9. doi: 10.1111/j.1349-7006.2008.01021.x 19200259 PMC11158820

[92] Swystun LL Mukherjee S Liaw PC . Breast cancer chemotherapy induces the release of cell‐free DNA, a novel procoagulant stimulus. J Thromb Haemostasis. (2011) 9:2313–21. doi: 10.1111/j.1538-7836.2011.04465.x 21838758

[93] Ma G Wang J Huang H Han X Xu J Veeramootoo JS . Identification of the plasma total cfDNA level before and after chemotherapy as an indicator of the neoadjuvant chemotherapy response in locally advanced breast cancer. Cancer Med. (2020) 9:2271–82. doi: 10.1002/cam4.2906 32017472 PMC7131846

[94] Zhang X Huang P Chen H Yang C Yang X Liu Y . Chemotherapy-induced CA-repeat DNA fragments in breast cancer trigger antitumor immune responses. Nat Immunol. (2025) 26:1931–45. doi: 10.1038/s41590-025-02289-2 41023478

[95] Alharbi AS Sapkota S Zhang Z Jin R Rupasinghe E Jayasekara WSN . 2′-O-methyl-guanosine RNA fragments antagonize TLR7 and TLR8 to limit autoimmunity. Nat Immunol. (2026) 27:762–75. doi: 10.1038/s41590-026-02429-2 41667621 PMC13043311

[96] Li G Zhao X Zheng Z Zhang H Wu Y Shen Y . cGAS-STING pathway mediates activation of dendritic cell sensing of immunogenic tumors. Cell Mol Life Sci. (2024) 81:149. doi: 10.1007/s00018-024-05191-6 38512518 PMC10957617

[97] Kang TH Mao CP Kim YS Kim TW Yang A Lam B . TLR9 acts as a sensor for tumor-released DNA to modulate anti-tumor immunity after chemotherapy. J Immunother Cancer. (2019) 7:260. doi: 10.1186/s40425-019-0738-2 31619293 PMC6794732

[98] Mouchess ML Arpaia N Souza G Barbalat R Ewald SE Lau L . Transmembrane mutations in Toll-like receptor 9 bypass the requirement for ectodomain proteolysis and induce fatal inflammation. Immunity. (2011) 35:721–32. doi: 10.1016/j.immuni.2011.10.009 22078797 PMC3230302

[99] Deane JA Pisitkun P Barrett RS Feigenbaum L Town T Ward JM . Control of TLR7 expression is essential to restrict autoimmunity and dendritic cell expansion. Immunity. (2007) 27:801–10. doi: 10.1016/j.immuni.2007.09.009 17997333 PMC2706502

[100] Barton GM Kagan JC Medzhitov R . Intracellular localization of Toll-like receptor 9 prevents recognition of self DNA but facilitates access to viral DNA. Nat Immunol. (2006) 7:49–56. doi: 10.1038/ni1280 16341217

[101] Lande R Gregorio J Facchinetti V Chatterjee B Wang YH Homey B . Plasmacytoid dendritic cells sense self-DNA coupled with antimicrobial peptide. Nature. (2007) 449:564–9. doi: 10.1038/nature06116 17873860

[102] Badal D Dayal D Singh G Sachdeva N . Role of DNA-LL37 complexes in the activation of plasmacytoid dendritic cells and monocytes in subjects with type 1 diabetes. Sci Rep. (2020) 10:8896. doi: 10.1038/s41598-020-65851-y 32483133 PMC7264208

[103] Diana J Simoni Y Furio L Beaudoin L Agerberth B Barrat F . Crosstalk between neutrophils, B-1a cells and plasmacytoid dendritic cells initiates autoimmune diabetes. Nat Med. (2013) 19:65–73. doi: 10.1038/nm.3042 23242473

[104] Ganguly D Chamilos G Lande R Gregorio J Meller S Facchinetti V . Self-RNA–antimicrobial peptide complexes activate human dendritic cells through TLR7 and TLR8. J Exp Med. (2009) 206:1983–94. doi: 10.1084/jem.20090480 19703986 PMC2737167

[105] Duan Z Fang Y Sun Y Luan N Chen X Chen M . Antimicrobial peptide LL-37 forms complex with bacterial DNA to facilitate blood translocation of bacterial DNA and aggravate ulcerative colitis. Sci Bull. (2018) 63:1364–75. doi: 10.1016/j.scib.2018.09.014 36658908

[106] Scheuerer B Ernst M Dürrbaum-Landmann I Fleischer J Grage-Griebenow E Brandt E . The CXC-chemokine platelet factor 4 promotes monocyte survival and induces monocyte differentiation into macrophages. Blood. (2000) 95:1158–66. doi: 10.1182/blood.V95.4.1158.004k31_1158_1166 10666185

[107] van Bon L Affandi AJ Broen J Christmann RB Marijnissen RJ Stawski L . Proteome-wide analysis and CXCL4 as a biomarker in systemic sclerosis. N Engl J Med. (2014) 370:433–43. doi: 10.1056/NEJMoa1114576 24350901 PMC4040466

[108] Lande R Lee EY Palazzo R Marinari B Pietraforte I Santos GS . CXCL4 assembles DNA into liquid crystalline complexes to amplify TLR9-mediated interferon-α production in systemic sclerosis. Nat Commun. (2019) 10:1731. doi: 10.1038/s41467-019-09683-z 31043596 PMC6494823

[109] Peng L Zhu N Mao J Huang L Yang Y Zhou Z . Expression levels of CXCR4 and CXCL12 in patients with rheumatoid arthritis and its correlation with disease activity. Exp Ther Med. (2020) 20:1925–34. doi: 10.3892/etm.2020.8950 32782501 PMC7401245

[110] Meller S Di Domizio J Voo KS Friedrich HC Chamilos G Ganguly D . TH17 cells promote microbial killing and innate immune sensing of DNA via interleukin 26. Nat Immunol. (2015) 16:970–9. doi: 10.1038/ni.3211 26168081 PMC4776746

[111] Dambacher J Beigel F Zitzmann K De Toni EN Göke B Diepolder HM . The role of the novel Th17 cytokine IL-26 in intestinal inflammation. Gut. (2009) 58:1207–17. doi: 10.1136/gut.2007.130112 18483078

[112] Chen R Kang R Tang D . The mechanism of HMGB1 secretion and release. Exp Mol Med. (2022) 54:91–102. doi: 10.1038/s12276-022-00736-w 35217834 PMC8894452

[113] Dumitriu IE Baruah P Bianchi ME Manfredi AA Rovere-Querini P . Requirement of HMGB1 and RAGE for the maturation of human plasmacytoid dendritic cells. Eur J Immunol. (2005) 35:2184–90. doi: 10.1002/eji.200526066 15915542

[114] Tian J Avalos AM Mao SY Chen B Senthil K Wu H . Toll-like receptor 9–dependent activation by DNA-containing immune complexes is mediated by HMGB1 and RAGE. Nat Immunol. (2007) 8:487–96. doi: 10.1038/ni1457 17417641

[115] Ivanov S Dragoi AM Wang X Dallacosta C Louten J Musco G . A novel role for HMGB1 in TLR9-mediated inflammatory responses to CpG-DNA. Blood. (2007) 110:1970–81. doi: 10.1182/blood-2006-09-044776 17548579 PMC1976374

[116] Du Y Ah Kioon MD Laurent P Chaudhary V Pierides M Yang C . Chemokines form nanoparticles with DNA and can superinduce TLR-driven immune inflammation. J Exp Med. (2022) 219:e20212142. doi: 10.1084/jem.20212142 35640018 PMC9161158

[117] Fang K Yuan S Zhang X Zhang J Sun S Li X . Regulation of immunogenic cell death and potential applications in cancer therapy. Front Immunol. (2025) 16. doi: 10.3389/fimmu.2025.1571212 40207233 PMC11979251

[118] Trotter TN Shuptrine CW Tsao LC Marek RD Acharya C Wei JP . IL26, a noncanonical mediator of DNA inflammatory stimulation, promotes TNBC engraftment and progression in association with neutrophils. Cancer Res. (2020) 80:3088–100. doi: 10.1158/0008-5472.CAN-18-3825 32366475 PMC7415539

[119] Vandercappellen J Van Damme J Struyf S . The role of the CXC chemokines platelet factor-4 (CXCL4/PF-4) and its variant (CXCL4L1/PF-4var) in inflammation, angiogenesis and cancer. Cytokine Growth Factor Rev. (2011) 22:1–18. doi: 10.1016/j.cytogfr.2010.10.011 21111666

[120] Kang R Zhang Q Zeh HJ Lotze MT Tang D . HMGB1 in cancer: Good, bad, or both? Clin Cancer Res. (2013) 19:4046–57. doi: 10.1158/1078-0432.CCR-13-0495 23723299 PMC3732559

[121] Chuang CM Monie A Wu A Mao CP Hung CF . Treatment with LL-37 peptide enhances antitumor effects induced by CpG oligodeoxynucleotides against ovarian cancer. Hum Gene Ther. (2009) 20:303–13. doi: 10.1089/hum.2008.124 19272013 PMC2855250

[122] Popovic PJ DeMarco R Lotze MT Winikoff SE Bartlett DL Krieg AM . High mobility group B1 protein suppresses the human plasmacytoid dendritic cell response to TLR9 agonists1. J Immunol. (2006) 177:8701–7. doi: 10.4049/jimmunol.177.12.8701 17142771

[123] Demoulin S Herfs M Somja J Roncarati P Delvenne P Hubert P . HMGB1 secretion during cervical carcinogenesis promotes the acquisition of a tolerogenic functionality by plasmacytoid dendritic cells. Int J Cancer. (2015) 137:345–58. doi: 10.1002/ijc.29389 25492101

[124] de Mingo Pulido Á Hänggi K Celias DP Gardner A Li J Batista-Bittencourt B . The inhibitory receptor TIM-3 limits activation of the cGAS-STING pathway in intra-tumoral dendritic cells by suppressing extracellular DNA uptake. Immunity. (2021) 54:1154–1167.e7. doi: 10.1016/j.immuni.2021.04.019 33979578 PMC8192496

[125] Chiba S Baghdadi M Akiba H Yoshiyama H Kinoshita I Dosaka-Akita H . Tumor-infiltrating DCs suppress nucleic acid–mediated innate immune responses through interactions between the receptor TIM-3 and the alarmin HMGB1. Nat Immunol. (2012) 13:832–42. doi: 10.1038/ni.2376 22842346 PMC3622453

[126] Zhang Y Gao J Wang X Deng S Ye H Guan W . CXCL4 mediates tumor regrowth after chemotherapy by suppression of antitumor immunity. Cancer Biol Ther. (2015) 16:1775–83. doi: 10.1080/15384047.2015.1095404 26479470 PMC4847813

[127] Radisky ES . Extracellular proteolysis in cancer: Proteases, substrates, and mechanisms in tumor progression and metastasis. J Biol Chem. (2024) 300(6):107347. doi: 10.1016/j.jbc.2024.107347 38718867 PMC11170211

[128] Poli C Augusto JF Dauvé J Adam C Preisser L Larochette V . IL-26 confers proinflammatory properties to extracellular DNA. J Immunol. (2017) 198:3650–61. doi: 10.4049/jimmunol.1600594 28356384

[129] Wong A Bryzek D Dobosz E Scavenius C Svoboda P Rapala-Kozik M . A novel biological role for peptidyl-arginine deiminases: Citrullination of cathelicidin LL-37 controls the immunostimulatory potential of cell-free DNA. J Immunol. (2018) 200:2327–40. doi: 10.4049/jimmunol.1701391 29475987 PMC5860981

[130] Lande R Ganguly D Facchinetti V Frasca L Conrad C Gregorio J . Neutrophils activate plasmacytoid dendritic cells by releasing self-DNA–peptide complexes in systemic lupus erythematosus. Sci Transl Med. (2011) 3:73ra19. doi: 10.1126/scitranslmed.3001180 21389263 PMC3399524

[131] Shao BZ Yao Y Li JP Chai NL Linghu EQ . The role of neutrophil extracellular traps in cancer. Front Oncol. (2021) 11. doi: 10.3389/fonc.2021.714357 34476216 PMC8406742

[132] Wang X Mayorga-Flores M Bien KG Bailey AO Iwahara J . DNA-mediated proteolysis by neutrophil elastase enhances binding activities of the HMGB1 protein. J Biol Chem. (2022) 298:102577. doi: 10.1016/j.jbc.2022.102577 36220391 PMC9664404

[133] Yun TJ Igarashi S Zhao H Perez OA Pereira MR Zorn E . Human plasmacytoid dendritic cells mount a distinct antiviral response to virus-infected cells. Sci Immunol. (2021) 6:eabc7302. doi: 10.1126/sciimmunol.abc7302 33811059 PMC8221820

